# Effect of printing orientation and resin thickness on flexural strength of direct 3D-printed aligners

**DOI:** 10.1186/s12903-025-05556-8

**Published:** 2025-02-14

**Authors:** Ahmed S. Khalil, Abbas R. Zaher

**Affiliations:** Department of Orthodontics, Faculty of Dentistry, Alexandria University, Champollion St, P.O. Box: 21521, Alexandria, Egypt

**Keywords:** Clear aligners, Direct printed aligners, Printing orientation, Mechanical properties, Flexural strength

## Abstract

**Background:**

Direct 3D-printed aligners served as a breakthrough era in clear aligner fabrication. Yet, there is a scarcity of studies evaluating their mechanical properties. The aim of this study was to compare direct 3D-printed aligners derived from different printing orientations (vertically, horizontally, 30, and 45 degrees) and thickness (0.5 and 0.7 mm) in terms of flexural strength.

**Methods:**

This laboratory-based comparative study utilized 96 aligner flat specimens. They were designed, supported, and directly printed using shape memory resin, then randomly allocated into 8 groups. Group 1 (A, B, C, and D): 0.5 mm thickness printed vertically, horizontally, 30, and 45 degrees, respectively. Group 2 (A, B, C, and D): 0.7 mm thickness printed vertically, horizontally, 30, and 45 degrees, respectively. Each aligner specimen was placed on a custom-made bending jig, with the whole setup enclosed in a temperature-controlled water bath. Three-point bending test was performed using a universal testing machine, and the resulting force was recorded. Statistical analysis was performed using Student t-test for resin thickness comparison and one-way ANOVA with Tukey post-hoc test for comparison between printing orientations. Statistical significance was set at *p* ≤ 0.05.

**Results:**

No statistically significant differences were found between vertically, horizontally, 30, and 45 degrees printed aligner specimens. Aligner specimens of 0.7 mm thickness demonstrated significantly higher flexural strength values compared to those of 0.5 mm thickness.

**Conclusions:**

Printing orientation did not alter the flexural strength of the direct 3D-printed aligner flat specimens, regardless of whether they were printed vertically, horizontally, or at angles of 30 or 45 degrees relative to the printer build plate. Additionally, specimens with a thickness of 0.7 mm exhibited higher bending resistance compared to those with a thickness of 0.5 mm.

## Background

The advent of clear aligners was a consequence of the increasing demand of highly esthetic treatment option among adult patients [1, 2]. Aligners have continually evolved in an attempt to meet the desire of orthodontic treatment with less visible appliances [3]. Furthermore, the feasibility of scanners and 3D printers combined with the advancement in technology have made a substantial surge in utilizing aligners as a highly convenient treatment modality relative to the conventional fixed appliances [4–7]. Traditional indirect fabrication of aligners involves 3D printing of models and consequently molding thermoformed plastic sheets over them [8]. Yet, this time consuming procedure coupled with an increased cost and potential geometric inaccuracies remained an inevitable challenge [9, 10]. Adoption of direct 3D printed aligners served as a breakthrough era in clear aligner fabrication. This contributed to eradication of intermediate steps and thereby diminished waste [11]. Stereolithographic 3D printing technology has found various applications in fabrication of occlusal splints, surgical guides, and nasoalveolar molding [12, 13]. A variety of orthodontic auxiliaries have also been manufactured in the contemporary era using 3D printers [14, 15]. 3D printed resin, when appropriately oriented, has exhibited certain anisotropic characteristics [16]. Utilizing different aligner thickness via direct 3D printing might broaden the possibility of superior mechanical performance [17, 18]. In essence, direct 3D printed aligners with altered thickness might pertain to generate force magnitudes closer to ideal range [19]. Interestingly, direct 3D printed aligners exhibited superior performance with a predictable and consistent force levels in comparison to thermoformed aligners [19].

Additionally, the Tera Harz TC-85 photocurable resin (Graphy, Seoul, South Korea) particularly displayed higher dimensional accuracy along with directional forces [18]. Using pressure columns in conjunction with direct printed aligner yielded promising results in extruding incisors in absence of attachments [20]. In the clinical setting, reducing appliance visibility essentially influence patient preference [21]. The pronounced concerns of esthetics for most patients imply less visibility [22]. The ability of the direct printed aligners to accomplish challenging orthodontic movements concomitant with avoidance of attachments would be a privilege [20].

Strength refers to a material’s capacity to withstand stress without undergoing plastic deformation or fracturing [23]. Flexural strength, a critical mechanical property for dental materials, serves as a fundamental parameter to evaluate their clinical performance [24]. In addition to the type of 3D printing employed, various factors were found to influence the flexural strength of the 3D-printed resin. This included printing direction, post-polymerization time and temperature, as well as the layer thickness used during printing [25, 26]. Additionally, a recent systematic review highlighted that the resin composition itself could significantly contribute to the flexural strength of the material [27]. 

Despite the rapid emergence of direct 3D-printed aligners, there is a scarcity of studies examining their mechanical properties compared to conventional thermoformed aligners. Understanding the fundamental properties of this innovative technology will provide clinicians with valuable insights into its clinical performance. This knowledge allows orthodontists to make well-informed choices regarding the printing orientation that best meets the optimal mechanical properties. Among these properties, flexural strength is particularly important, as it directly influences the forces exerted by aligners during treatment, thereby impacting the overall treatment outcomes. Hence, the aim of the present study was to compare direct 3D-printed aligner specimens derived from different printing orientations and thickness in terms of flexural strength. The null hypothesis was that there is no difference of flexural strength regardless the printing orientation or thickness employed.

## Methods

### Specimen fabrication

Power analysis showed that a minimum of 10 specimens per group were required to detect a clinically meaningful difference between the groups at a test power of 90% and a level of significance of 0.05 [28]. A total of 12 specimens per group were employed to cater for any potential damage during the study. The study composed of 8 groups with four distinct printing orientations on the printing platform, used for specimens with thickness of 0.5 and 0.7 mm. Experimental groups were as follows;

- Group 1 A: 0.5 mm specimen thickness printed vertically.
- Group 1B: 0.5 mm specimen thickness printed horizontally.
- Group 1 C: 0.5 mm specimen thickness printed at 30 degrees.
- Group 1D: 0.5 mm specimen thickness printed at 45 degrees.
- Group 2 A: 0.7 mm specimen thickness printed vertically.
- Group 2B: 0.7 mm specimen thickness printed horizontally.
- Group 2 C: 0.7 mm specimen thickness printed at 30 degrees.
- Group 2D: 0.7 mm specimen thickness printed at 45 degrees.

**Fig. 1:**
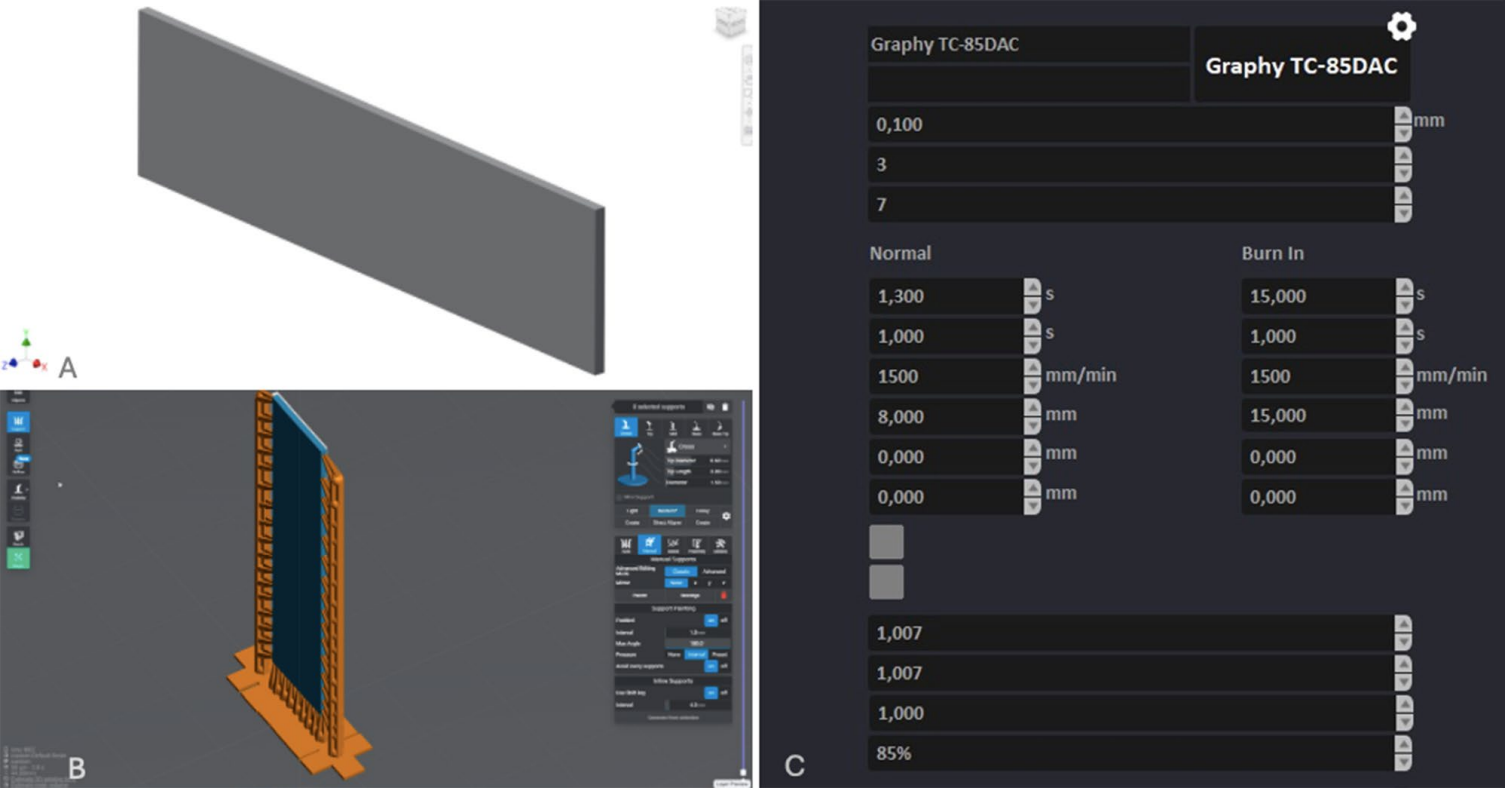
**A** Aligner specimen designed, **B** Supports’ generation, **C** Printing parameters employed

**Fig. 2:**
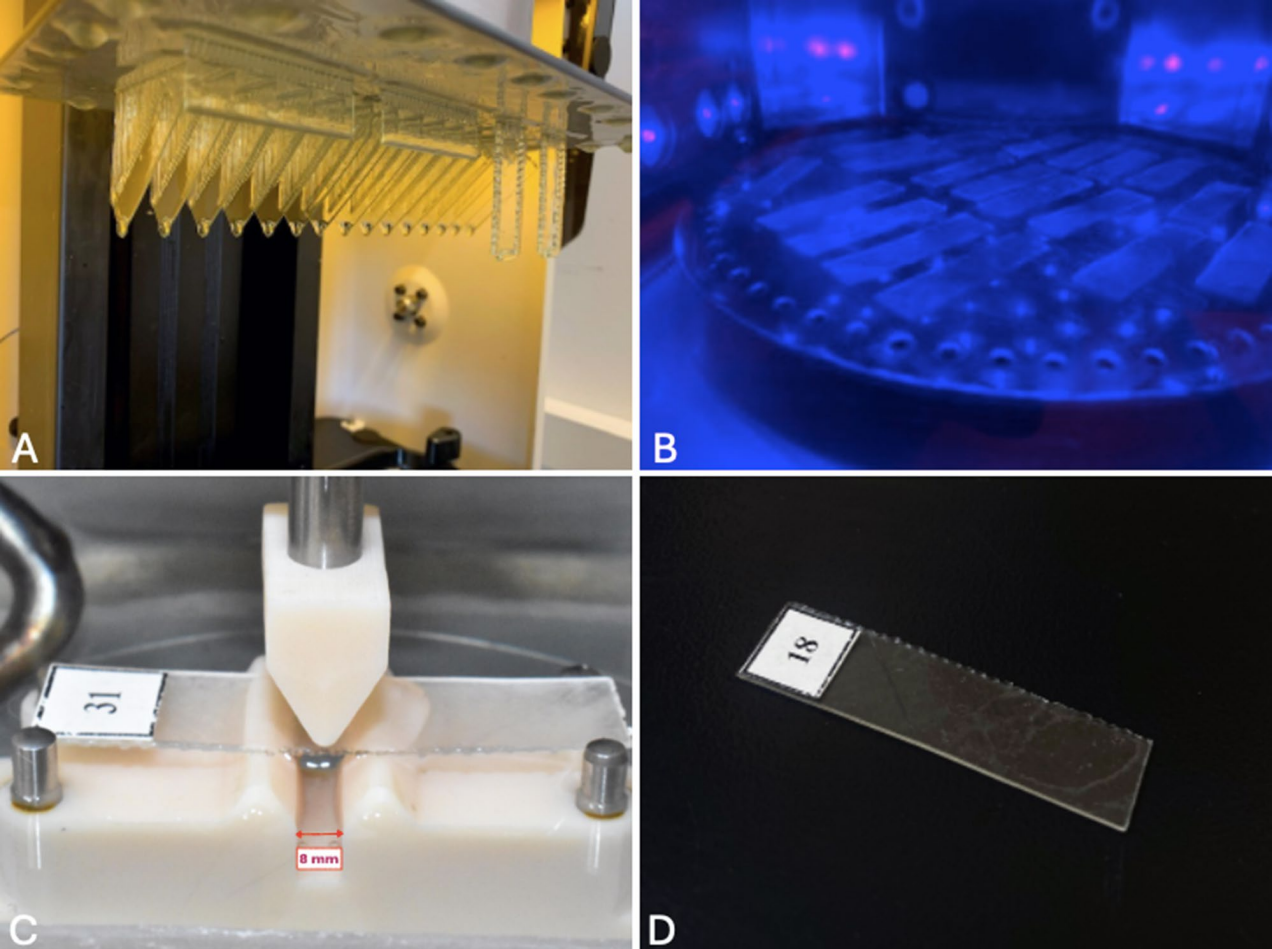
**A** Specimens after printing, **B** Curing of specimens using nitrogen chamber, **C** Three-point bending jig, **D** Specimen after removal of the supports

Ninety-sixflat aligner specimens of 40 mm length, 10 mm width, and thickness of either 0.5–0.7 mm according to their group assignment, were designed using Inventor CAD software (Autodesk, California, USA) and exported as stereolithography (STL) format files (Fig. 1A). The STL files were then imported into Lychee Slicer Pro Resin and Filament software (Mango 3D, Merignac, France) to create supports (Fig. 1B). The designs were subsequently sliced using Uniz software (Uniz, California, USA) and printed using NBEE printer (Uniz, California, USA) either vertically, horizontally, at an angle of 30 or 45 degrees relative to the printer build plate with 100-mm layer height following the manufacturer’s instructions (Fig. 1C). The printing resin used was Tera Harz TC-85 DAC, a photocurable shape memory aliphatic vinyl ester-urethane resin (Graphy, Seoul, South Korea) [29]. After printing, aligner flat specimens, with their supports, were removed from the printer build plate (Fig. 2A) and subjected to centrifugation for 6 min to remove any residual uncured resin using Tera Harz Spinner THS centrifuge (Graphy, Seoul, South Korea) of 500 rpm with a power and frequency of 220 V and 60 Hz, respectively [30]. Subsequently, the supports were manually removed and the specimens were then cured using Tera Harz Cure THC (Graphy, Seoul, South Korea) oxygen-free nitrogen curing chamber for 20 min with a wavelength and output of 405 nm and 200 W, respectively [31] (Fig. 2B). Finally, ultrasonic cleaning of the specimen was done at 80 °C for 1 min using Sonorex Super RK 103 H (Bandelin, Berlin, Germany).

### Bending jig fabrication

A three-point bending jig, made of 8 mm span length between the lateral supports, was designed using Inventor CAD, sliced with Envision One RP Slicer (ETEC, Gladbeck, Germany), then printed using the same printing process employed with the aligner flat specimens. The lateral supports had a 1 mm radius and were positioned on a 60 degrees wedge (Fig. 2C).

### The experimental setup

The experimental apparatus consisted of aligner specimens, bending jig, water bath chamber (20 × 20 × 10 cm), electric heater rod, mercury-in-glass, digital thermometer, and universal testing machine (Tinius Oslen, TMC, Horsham, PA, USA).

### Three-point bending test scheme

**Fig. 3:**
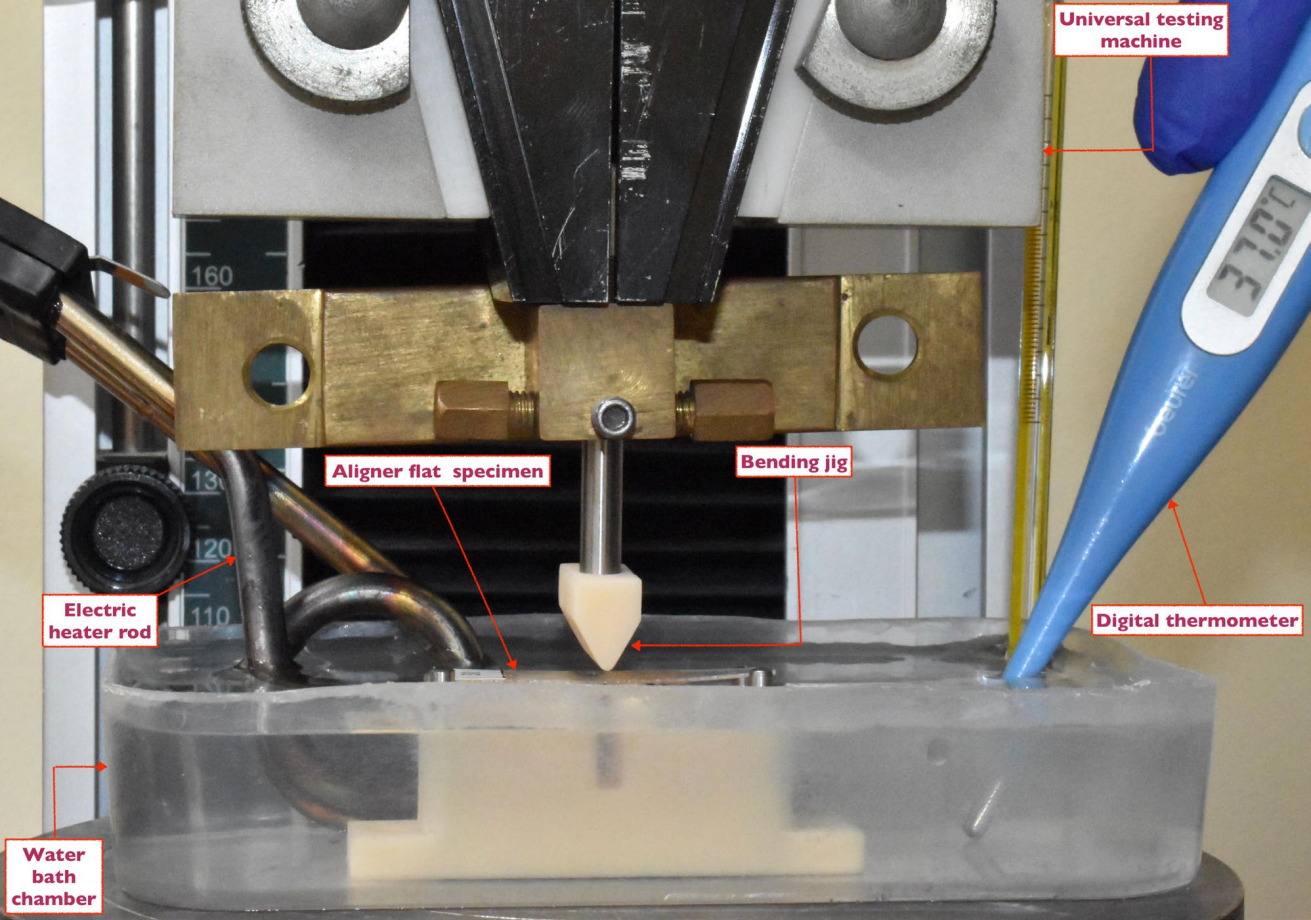
Experiment’s setup: aligner flat specimen placed on a custom-made bending jig, with the whole setup enclosed in a temperature-controlled water bath

Prior to the test, each specimen was designated a number identifier using a waterproof label (Fig. 2D). Numbers from 1 to 96 were assigned using a computer-generated Microsoft Office Excel 2013 sheet. An individual examiner who had received training and calibration conducted the three-point bending test with the guidance of a specialist technician. Each aligner specimen was placed on the bending jig, with the whole setup enclosed in a temperature-controlled water bath chamber using thermal heater to sustain a temperature of 37 °C, effectively simulating intraoral environment [32] (Fig. 3).

A universal testing machine was employed at a crosshead speed of 1 mm/min to a maximum deflection of 3 mm for three-point-bending of the specimens according to ISO 20795-2 and the resulting force was recorded by LabView 8.5 software [33]. (National Instruments Corporation, Austin, USA)

The flexural strength was calculated using the following equation [34]:

$$\:Flexural\:Strength=\frac{3PL}{2B{D}^{2}}$$

**Fig. 4:**
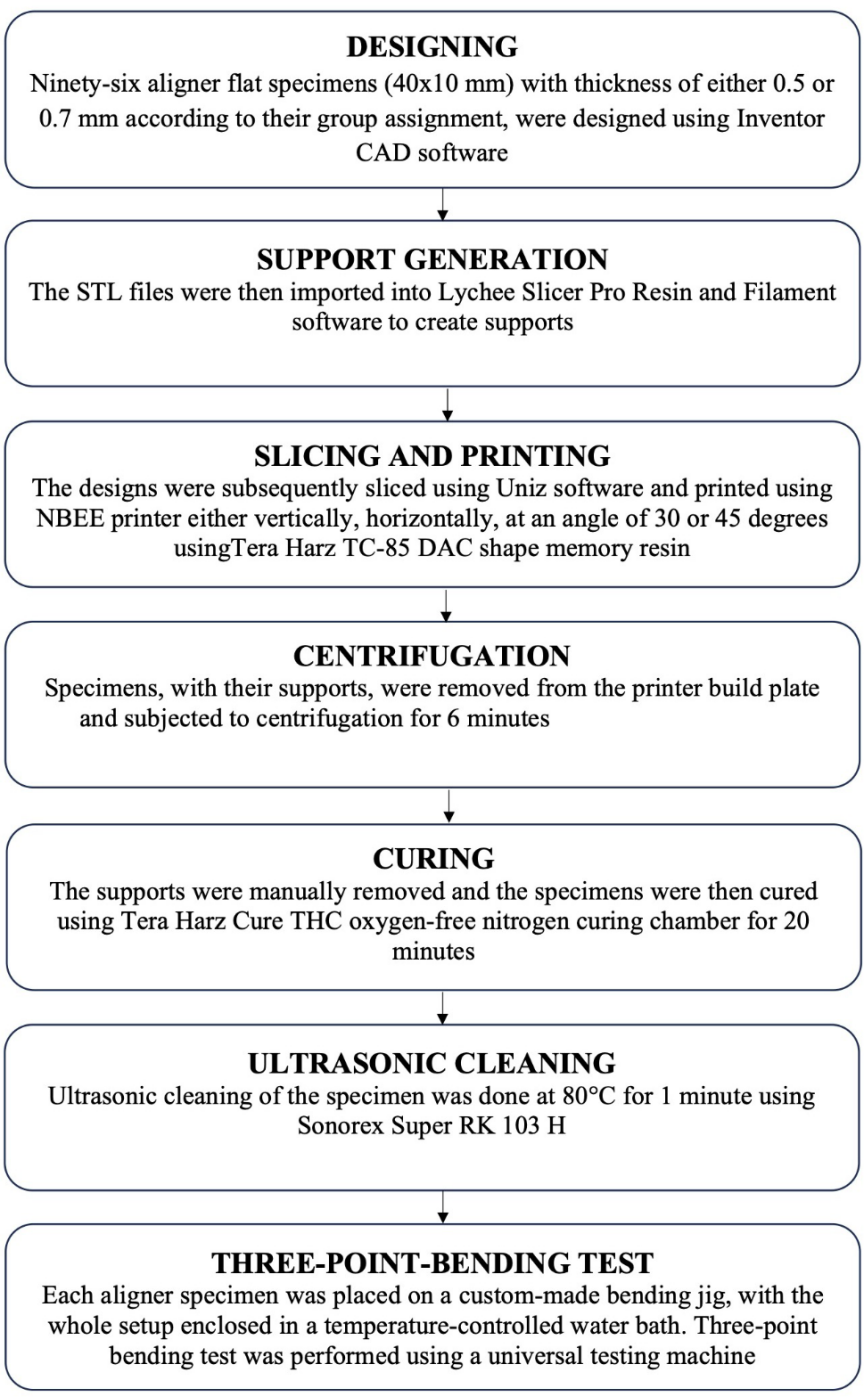
Study flow chart

Where P was load, L is span length, B is width of specimen, D is thickness of specimen. A detailed flow chart is depicted in (Fig. 4).

### Statistical analysis

Flexural strength values were described using mean and standard deviation. The Shapiro-Wilk test was used to evaluate data distribution normality. Student t-test was used to compare between 0.5 and 0.7 thickness while F-test (ANOVA) was used for flexural strength to compare between more than two groups. Post Hoc test (Tukey) was used for pairwise comparisons. Significance of the obtained results was set at the 5% level (*P* ≤ 0.05). All analysis were performed using IBM SPSS software package version 20.0. Visualization was done using canva (www.canva.com).

## Results

Descriptive analysis and analytical statistics of flexural strength values are depicted in Table 1. The results of flexural strength showed no statistically significant difference between vertically, horizontally, 30, and 45 degrees printed aligner flat specimens. A statistically significant higher flexural strength values was found in aligner specimens of 0.7 mm compared to 0.5 mm thickness.

The mean flexural strength was 55.63, 57.78, 58.17, and 55.22 megapascal (MPa) for 0.5 mm specimen thickness printed vertically, horizontally, at 30, and at 45 degrees, respectively. Finally, the mean flexural strength was 64.83, 67.14, 65.19, and 63.43 MPa for 0.7 mm specimen thickness printed vertically, horizontally, at 30, and at 45 degrees, respectively.

**Table 1:** Comparison between the test groups

Flexural strength (MPa)	Group A (*n* = 12)	Group B (*n* = 12)	Group C (*n* = 12)	Group D (*n* = 12)	F	*P*
Thickness (mm) 0.5	55.63 ± 2.46	57.78 ± 2.24	58.17 ± 6.96	55.22 ± 1.71	1.708	0.179
Thickness (mm) 0.7	64.83 ± 5.20	67.14 ± 4.07	65.19 ± 4.47	63.43 ± 3.52	1.479	0.233
T	5.532^*^	6.985^*^	2.938^*^	7.279^*^		
P_1_	< 0.001^*^	< 0.001^*^	0.008^*^	< 0.001^*^		

Flexural strength values are presented as the mean ± standard deviation

MPa: megapascal

Group A: aligner flat specimens printed vertically relative to the printer build plate, Group B: horizontally, Group C: at angle of 30 degrees, Group D: at angle of 45 degrees

t: Student t-test

F: F for One way ANOVA test

Pairwise comparison between each 2 groups was done using Post Hoc Test (Tukey)

P: P value for comparing between test groups

P_1_: P value for comparing between 0.5 and 0.7 thickness

*: Statistical significant difference

Statistical significance was considered at *P* ≤ 0.05

## Discussion

This laboratory-based study was conducted to compare different printing orientations. Besides the fact that direct printing of aligners is a relatively new technology in orthodontics with little evidence, comparing our findings with the results of previous studies was challenging due to variations in study designs, type of resin and printer used, and inconsistency in the methodology across the studies. Aligner flat specimens were virtually designed, supported, and directly printed at 4 different printing orientations. The use of oxygen-free nitrogen chamber was recommended by the manufacturer to ensure complete polymerization of the printed parts [31]. The presence of oxygen was found to compromise the transparency and mechanical properties and inhibit complete polymerization which subsequently raise the concern of health hazards due to the potential leaching of uncured urethane dimethacrylate monomer [35]. The primary advantage of employing three-point bending test is the simplified specimen geometry. Hence, this simplified testing setup enables assessment of individual factors affecting the force transmission of aligners. Consequently, setting up the test is relatively straightforward and inexpensive. That being said, studying the stiffness of aligners emphasizes the significance of adequate force application for tooth movement while avoiding excessive force that could potentially induce periodontal issues [36, 37]. 

In the present study, the null hypothesis could not be rejected as we found no statistically significant difference of bending strength regardless the printing orientation employed. This implies that bending strength of the directly printed aligner specimen was not affected by the printing orientation employed. However, it has long been postulated that printing horizontally tends to result in reduced printing duration and a more need for supports [38]. This is translated into an increased amount of resin. The increased number of supports might also require a longer time for post-processing support removal. Nevertheless, vertical printing orientation was recommended in a study due to the reduced need of number of supports [39]. Aligners specimen printed at 30 and 45 degrees provided an intermediate choice between these printed horizontally and vertically. It is worth mentioning that the further the aligners extend from build platform, the greater printing layers would be required. This translates into increased printing duration and greater likelihood of printing failure due to the flexible nature of the printed and the encountered suction forces during printing [28]. 

The selection of specimens with 40 mm length, and 10 mm width was based on the findings of a previous study [40]. Also, the use of specimens with thickness of 0.5 mm was selected on the study of Lee et al. [41] While it was expected that specimens with greater thickness would exhibit higher bending resistance, we particularly compared 0.5 and 0.7 thickness based on the fact that increasing the thickness of thermoformed retainers results in reduced breakage [42]. In the present study, the highest flexural strength values were found in the specimen of 0.7 mm thickness. This finding is in line with a previous study, where increased aligner thickness exhibited higher bending resistance [43]. A noteworthy finding of a previous study was a 10 times higher relaxation index of directly printed aligners compared with the conventional thermoformed aligners [44]. This raises concerns about the potential level of relaxation that directly printed aligners might experience in clinical conditions. Interestingly, significant difference was found for mechanical properties of directly printed aligners derived from five different 3D printers despite using the same resin and post-processing steps [45]. The variation in mechanical properties was explained by the diverse technology employed to flash light on resin.

In our experiment, a speed of 1 mm/min a maximum deflection of 3 mm was selected because direct printed aligner specimen is prone to crack when subjected to greater deflection [40, 43]. The span length between the lateral supports was 8 mm. A previous study found that 8 mm span length was superior to 16 mm. This was explained by the localized stress concentration on diminished aligner area [40]. Moreover, Elkholy et al. recommended using flat specimens for material testing. The rationale behind this was that the three-point bending test should utilize the most symmetrical form possible, a feature that direct printed aligners lack [40]. Remarkably, simulation of the complex 3D is hard to achieve and consequently flat specimen with its symmetric and simple form was employed.

Despite the promising findings of directly printed aligner reported in previous research [32, 41, 46, 47], there are multiple aspects that still necessitate further investigation. This includes testing printer types, printing atmosphere, postprocessing curing, and biocompatibility of the resin used prior to establishing it as a viable substitute to conventional thermoformed aligners.

### Limitations

There are several limitations with the in-vitro experimental study design, notably the lack of 3D complex morphology of aligners, simulated saliva, and periodontal ligament representation along with the inherent anisotropic characteristics of 3D-printed resin. Moreover, the absence of mastication forces diminishes the clinical relevance of the current study. Further studies are required to study the mechanical properties of aligners in a more clinically oriented model.

## Conclusion

Based on the results of the current study, we found no difference in the flexural strength values of the direct 3D-printed aligner flat specimens either printed vertically, horizontally, at angle of 30 or 45 degrees relative to the printer build plate. Overall, 0.7 mm thickness exhibited higher bending resistance. Further research on the mechanical properties of direct printed aligners presents a new realm for advancement in clear aligner field.

## Data Availability

The datasets used during the current study are available from the corresponding author on reasonable request. All data analyzed during this study are included in this published article in the form of tables and figures.

## Abbreviation

MPa: Megapascal
